# Improving the Performance of Electrotactile Brain–Computer Interface Using Machine Learning Methods on Multi-Channel Features of Somatosensory Event-Related Potentials

**DOI:** 10.3390/s24248048

**Published:** 2024-12-17

**Authors:** Marija Novičić, Olivera Djordjević, Vera Miler-Jerković, Ljubica Konstantinović, Andrej M. Savić

**Affiliations:** 1 School of Electrical Engineering, University of Belgrade, 11000 Belgrade, Serbia; 2 Faculty of Medicine, University of Belgrade, 11000 Belgrade, Serbia; 3 Clinic for Rehabilitation “Dr. Miroslav Zotović”, 11000 Belgrade, Serbia; 4 Innovation Center of the School of Electrical Engineering, University of Belgrade, 11000 Belgrade, Serbia

**Keywords:** brain–computer interface (BCI), somatosensory event-related potentials (sERPs), electrical stimulation, tactile attention, tactile BCI, feature selection, machine learning

## Abstract

Traditional tactile brain–computer interfaces (BCIs), particularly those based on steady-state somatosensory–evoked potentials, face challenges such as lower accuracy, reduced bit rates, and the need for spatially distant stimulation points. In contrast, using transient electrical stimuli offers a promising alternative for generating tactile BCI control signals: somatosensory event-related potentials (sERPs). This study aimed to optimize the performance of a novel electrotactile BCI by employing advanced feature extraction and machine learning techniques on sERP signals for the classification of users’ selective tactile attention. The experimental protocol involved ten healthy subjects performing a tactile attention task, with EEG signals recorded from five EEG channels over the sensory–motor cortex. We employed sequential forward selection (SFS) of features from temporal sERP waveforms of all EEG channels. We systematically tested classification performance using machine learning algorithms, including logistic regression, k-nearest neighbors, support vector machines, random forests, and artificial neural networks. We explored the effects of the number of stimuli required to obtain sERP features for classification and their influence on accuracy and information transfer rate. Our approach indicated significant improvements in classification accuracy compared to previous studies. We demonstrated that the number of stimuli for sERP generation can be reduced while increasing the information transfer rate without a statistically significant decrease in classification accuracy. In the case of the support vector machine classifier, we achieved a mean accuracy over 90% for 10 electrical stimuli, while for 6 stimuli, the accuracy decreased by less than 7%, and the information transfer rate increased by 60%. This research advances methods for tactile BCI control based on event-related potentials. This work is significant since tactile stimulation is an understudied modality for BCI control, and electrically induced sERPs are the least studied control signals in reactive BCIs. Exploring and optimizing the parameters of sERP elicitation, as well as feature extraction and classification methods, is crucial for addressing the accuracy versus speed trade-off in various assistive BCI applications where the tactile modality may have added value.

## 1. Introduction

Brain–computer interfaces (BCIs) provide control of external devices such as assistive systems by recognizing patterns in brain activity [1]. The most common way to record brain signals in BCI applications is a non-invasive technique called electroencephalography (EEG) [2]. Various EEG features can be used to generate control signals in a BCI system, such as oscillatory brain activity, event-related potentials (ERPs), steady-state evoked potentials (SSEPs), and slow cortical activity [3]. With the ERP and SSEP paradigms, changes in brain activity are generated with external stimuli, categorizing them as “reactive” BCI systems [4].

Based on the type of external stimuli, reactive BCI systems are divided into visual [5,6], auditory [7,8], and tactile [9]. Tactile BCI prototypes, in addition to including a device for measuring brain activity and a computer, also require a somatosensory stimulation device, which complicates the system’s setup. This complexity is one reason why these systems are less frequently mentioned in the literature compared to other reactive BCI systems relying on visual or auditory modality and thus requiring only a light or sound source [9]. External stimuli in tactile BCIs can be vibration or electrical somatosensory stimulation [10,11,12]. Vibration stimulation is often preferred over electrical stimulation in tactile BCIs due to its comfort, ease of implementation, and effectiveness. Somatosensory stimulation can elicit different responses in brain activity that can be used as control signals for BCIs, including somatosensory P300, event-related desynchronization (ERD), or somatosensory-evoked potentials (SEPs).

SEPs, which are elicited by electrically stimulating peripheral nerves, provide insight into neural activation along the somatosensory pathways [13]. In the context of tactile BCIs, SEPs are recorded at the cortical level. The rate of stimulation can be modulated to produce either transient or steady-state responses. Transient stimuli result in cortical SEP waveforms characterized by a series of signal components [14]. Conversely, when stimulation is continuous and the interval between stimuli is short, the somatosensory system does not return to its idle state. This leads to the generation of steady-state somatosensory-evoked potentials (SSSEPs) [15]. SSSEP-based control encounters several challenges, including diminished accuracy for tactile modalities and lower bit rates. Even with these drawbacks, SSSEP-based control is investigated in numerous studies [14].

When stimulation rate is set to evoke transient SEPs at the cortical level concurrently with a cognitive task involving focused attention on the stimulus, elicited responses are termed—somatosensory event-related potentials (sERPs). Those signals are a useful tool in basic research for investigating the spatiotemporal dynamics of neural processes related to selective tactile attention [16].

The use of tactile stimulation combined with transient responses (evoked or event-related potentials) as a BCI control signal has been investigated on a much smaller scale compared to SSSEPs, while the electrically induced transient responses were previously reported only in two studies in the form of electrically induced P300 responses [11,12]. These studies utilize the concept of the oddball paradigm to elicit a somatosensory P300 response with a low-probability target stimulus.

We recently proposed a novel sERP-based BCI control paradigm with the same (50%) probability of target and distractor stimuli while the user is performing a cognitive task—selective tactile attention focus [17]. In our initial studies [18,19], using a single-channel approach with an information transfer rate (ITR) of 4.29 bits per minute (bpm), we achieved accuracies ranging from 75.1% to 88.1% in healthy subjects and between 70% and 100% in subacute stroke survivors.

These results proved to be satisfactory for the initial application of our system for sensory training in subacute stroke; however, the assistive applications, such as BCI-based communication systems for severely disabled users, require improvements in ITR and accuracy. This paper focuses on optimizing the performance of our novel sERP-based BCI based on a combination of multi-channel EEG features and state-of-the-art feature extraction and machine learning approaches. This work is significant not only in the context of our novel sERP-based control and can be applied to any reactive BCI based on evoked or event-related potentials. The main goal of this study was to optimize the feature extraction and classification procedures for increasing the accuracy and ITR. This study aims to provide the following:

1. A method for feature selection based on multi-EEG-channel features.
2. A comparison of several systematically tested and optimized machine learning algorithms for classification of tactile attention.
3. The detailed exploration of the ITR vs. accuracy trade-off, i.e., the possibility of reduction in the number of single evoked responses to increase the ITR while sustaining satisfactory accuracy.

Our main hypothesis was that a multi-channel approach, novel feature selection, and machine learning methods can improve classification accuracy and the ITR of the BCI system based on sERPs. This work is significant because it deals with a recently introduced electrotactile BCI control paradigm, attempting to broaden its application range from restorative to assistive applications, which have higher performance requirements. We aimed to prove that our methods for BCI control signal generation, feature extraction, and classification could outperform the limited number of previously described tactile BCI prototypes and introduce improvements beyond the state-of-the-art in EEG-based tactile BCIs.

## 2. Materials and Methods

### 2.1. Subjects

In this study, the data of 10 healthy subjects (9 male, 1 female, average age 25.4 ± 2.91) was analyzed. Subjects were without history of neurological disorders and with normal or corrected-to-normal vision. The subjects had no previous experience with EEG recordings and were completely naive about the task. All the participants were informed about study procedures and signed a written informed consent form before participating. This study was approved by the local ethics committee and is in accordance with ethical guidelines defined by the Declaration of Helsinki.

### 2.2. Instrumentation and Experimental Setup

An eight-channel, current-controlled electrical stimulator, MOTIMOVE (3F–Fit Fabricando Faber, Belgrade, Serbia), was used for electrical stimulation (ES). This stimulator is fully programmable, allowing independent control of stimulation parameters (pulse width, pulse amplitude, and stimulation frequency) for each channel by sending commands from a PC via a USB port in real time. The stimulator is battery powered and fully isolated from the main power supply. In this study, two stimulation channels were employed, each with two active electrodes of 1 cm diameter placed on the dorsal (D location) and volar (V location) surfaces of the right forearm. A common indifferent electrode, 2.5 cm in diameter, was positioned on the volar aspect of the right wrist. In our previous publication [18], we provided a detailed explanation of the electrical stimulator setup and its fine-tuning. The stimuli used in this study were single, compensated biphasic pulses with exponential discharge. The current pulse duration was 0.25 ms in the active phase while the inter-pulse interval was set to 750 ms.

The EEG signals were recorded using the g.USBamp amplifier (g.tec GmbH, Schiedlberg, Austria). Six active electrodes (g.GAMMAcap2 connected to g.GAMMAbox, g.tec GmbH, Austria) were positioned at standard 10–20 locations: C3, Cz, C4, CP3, P3, and Fp1. The reference electrode was placed on the left earlobe, and AFz served as the ground location. The signal from the Fp1 electrode was specifically utilized to detect ocular artifacts. The amplifier was configured to use embedded notch filtering with a cut-off frequency of 50 Hz.

### 2.3. Experimental Protocol

For signal acquisition and stimulation control, an application was developed in the MATLAB (MathWorks Inc., Natick, MA, USA) programming environment. This application featured a graphical user interface (GUI) that allowed visualization of EEG signals and control over parameters of the electrical stimulator.

The participants were seated comfortably approximately 1 m away from a computer screen. At the beginning of the experiment, all EEG and ES electrodes were positioned, the quality of EEG signals was verified, and stimulation amplitudes for each channel were set. The motor threshold for each electrical stimulation channel was separately determined by gradually increasing the pulse amplitude from 5 mA in 1 mA increments. Once the motor stimulation elicited muscle twitch responses at each location, the stimulation amplitude decreased by 1 mA below the motor threshold. Subsequently, subjective stimulation sensations at two identified hotspots were balanced by decreasing the stimulation amplitude at the location that induced the stronger sensory response reported by the participant. The objective was to achieve the most similar subjective sensation produced by electrical stimulation at both sites.

The experiment comprised six blocks. Each block was divided into five sub-blocks interspersed with short pauses. Within each sub-block, 60 stimuli were delivered in a sequential, pseudorandomized order to D and V locations, with approximately 25–35 stimuli per location. A constraint was applied during randomization to ensure that no more than three consecutive stimuli were presented at the same location. Participants were instructed to attend to the target location by silently counting the number of stimuli delivered to that target location. The experimental condition (attended D or attended V location) alternately switched between blocks. During inter-block pauses, the subject reported their count of target stimuli. The subjects were instructed to limit body movement and to fix their gaze on a fixation cross at the center of the screen in order to minimize ocular movement. The total number of stimuli delivered per subject was 1800, that is, 900 per stimulus location (D and V).

### 2.4. Data Processing

EEG signals were processed offline in the MATLAB programming environment using custom scripts for signal processing and sERP extractions. The signals were filtered using a second-order zero-phased Butterworth band-pass filter with cut-off frequencies of 0.1 and 25 Hz for five EEG channels and 1 and 10 Hz for the Fp1 channel.

The EEG was segmented into 650 ms epochs, containing a 100 ms prestimulus baseline and a 550 ms poststimulus interval. Baseline of each epoch was corrected by subtracting the mean value of baseline from the poststimulus interval for each channel. Subsequently, epochs containing blink or movement artifacts were identified and removed from further analysis. The epochs were rejected by applying the threshold of 50 µV for all EEG channels and a threshold of 80 µV on the Fp1 channel. If the absolute value of either of the channels exceeded the designated threshold, that epoch was rejected from further analysis. Finally, 500 ms (600 signal samples) trials were extracted from epochs that correspond to the 50 to 550 ms poststimulus interval.

Trials were categorized into four sERP waveform clusters derived from two stimulation locations (D or V location) and two experimental conditions (attended D or attended V location) as follows:

1. Attended D location with stimulus delivered to D (ADSD);
2. Attended D location with stimulus delivered to V (ADSV);
3. Attended V location with stimulus delivered to D (AVSD);
4. Attended V location with stimulus delivered to V (AVSV).

In each subject, every cluster included over 400 individual sERPs after eliminating artifacts. On average, 39.17 ± 34.9 trials were rejected.

To extract relevant features for classification of users’ tactile attention, it was necessary to average individual sERP trials to enhance the signal-to-noise ratio. Given the importance of responsiveness in BCI systems, various numbers of trials to be averaged ($N_{avg}$), ranging from 2 to 10, were tested. Averaged sERP amplitudes were then downsampled by a factor of 8, resulting in 75 signal samples (amplitude values) per channel (equivalent to a new sample frequency of 150 Hz).

### 2.5. Feature Selection

In this study, we explored the multi-channel approach for forming feature vectors for classification. The methodology involved performing the feature selection process across all EEG channels collectively, thereby aggregating them into a single vector. For each sERP waveform cluster and each trial, five EEG channels were concatenated, resulting in a vector comprising 375 samples (5 channels × 75 signal samples). Subsequently, two input vectors were formed by joining the consecutive pairs of concatenated sERP waveforms: from clusters ADSD and ADSV for class AD and from AVSD and AVSV for class AV. Consequently, each input vector consisted of 750 samples, where the first half represented concatenated sERP amplitude values elicited by stimulation of the D location and the second half represented concatenated sERP responses elicited by stimulation of the V location.

To select relevant sERP amplitude features for further classification, we employed sequential forward selection (SFS) [20]. SFS is a wrapper method for feature selection that evaluates features using a specific machine learning algorithm to identify optimal subsets. We implemented a nested cross-validation approach where the outer 5-fold cross-validation (CV) was used for model evaluation, while feature selection was independently performed within each training fold using another 5-fold cross-validation. This nested structure ensures unbiased performance estimates and prevents data leakage.

Within each outer training fold, the SFS process started with an empty set of features and iteratively added features based on their contribution to the model’s performance. The maximum number of features to be selected was set to 40.

### 2.6. Classification Approaches

The objective of our study was to evaluate various machine learning algorithms for the classification of users’ tactile attention. Specifically, we compared five widely employed classifiers: logistic regression (LR), K-nearest neighbors (KNNs), support vector machine (SVM), random forest (RF), and artificial neural networks (ANNs).

The data was first split into 5 outer folds. For each outer-fold iteration, one-fold was held out as the test set and never used in any part of the model development process. Using only the remaining 4 folds (training set), we performed an inner 5-fold cross-validation to select features and optimize hyperparameters. This inner procedure was independently repeated for each outer fold, ensuring that feature selection and hyperparameter tuning were solely based on the training data of each fold. The final model for each outer fold was then evaluated on its respective held-out test data, and results were averaged across all outer folds.

For each classifier and outer fold of the 5-fold cross-validation (CV)

1. The training data is used for feature selection through SFS using nested 5-fold CV for feature evaluation and default classifier parameters.
2. Using the selected feature subset, hyperparameters are optimized through grid search using the same inner-fold splits.
3. The final model with selected features and optimized hyperparameters is evaluated on the held-out test data.

The classifier performance estimation algorithm with nested cross-validation is described in Algorithm 1.
**Algorithm 1.** Classifier performance estimation**Input:** Dataset X, maximum features k = 40 **Output:** Selected features Y, Average accuracy (Acc) 1. Split X into 5 outer folds 2. For each outer fold:        a. Set aside test data        b. On training data:                - Create inner 5-fold CV splits                - Initialize empty feature set: Y = {}                - While |Y| < k:                        * For each candidate feature f not in Y:                                - Using the created inner CV splits:                                        * Evaluate Acc(Y ∪ {f}) using default parameters                                - Select f* = argmax(Acc)                        * Add f* to Y if improves performance                - Using the created inner CV splits:                        * Perform hyperparameter tuning with selected features Y        c. Evaluate final model on outer fold test data 3. Return averaged accuracy (Acc) across outer folds

#### 2.6.1. Logistic Regression (LR)

Logistic regression [21] is a statistical technique commonly used in binary classification tasks to predict the probability of an event occurring based on predictor variables. The model assumes a linear relationship between the logit of the outcome and the predictors, employing maximum likelihood estimation to determine coefficients.

To reduce overfitting, regularization techniques were used during hyperparameter tuning. Specifically, two regularization techniques were evaluated: L1 (Lasso) and L2 (Ridge) regularization. For each of these techniques, regularization strength was optimized from the set of values [0.001,  0.01,  0.1,  1] to ensure robust generalization to unseen data. During the SFS procedure, the LR classifier was trained with L2 regularization and a regularization strength of 0.1.

#### 2.6.2. K-Nearest Neighbors (KNNs)

The KNN [22] is a non-parametric method commonly used for classification. The main idea of KNN is to determine the class of a data point by assessing the majority class among its K-closest neighbors in the feature space. The nearest data points are found using a distance measure, such as Euclidean distance. The choice of K significantly impacts the model’s performance. Smaller values of K add noise to the model’s outcome, while larger values increase computational complexity.

In our study, we systematically tested values of K within the range of 5 to 20 nearest neighbors to identify the optimal parameter setting that maximizes the algorithm’s classification accuracy and generalization capability. The value of K during the SFS procedure was set to 10.

#### 2.6.3. Support Vector Machine (SVM)

Support vector machines are prominent supervised learning models, grounded in statistical learning theory [23]. Their fundamental principle involves finding an optimal hyperplane that maximizes the margin between data points in a high-dimensional feature space. SVMs are particularly valuable in scenarios where data are not linearly separable. By transforming inputs using kernel functions, it is possible to create a nonlinear separation hyperplane in the original feature space.

Two types of kernel functions, linear and Gaussian radial basis function (RBF), were tested. For the RBF kernel function, we optimized the value of γ from the set of values [0.001,  0.01,  0.1,  1]. Additionally, to reduce overfitting, regularization was incorporated by optimizing the regularization coefficient C from the set of values [0.1,  1,  10,  100].

#### 2.6.4. Random Forest (RF)

Random forest is a powerful ensemble learning method widely employed in supervised learning tasks [24]. This technique operates by constructing multiple decision trees during training and combining their predictions to determine the final class. The key strength of the RF algorithm is that it reduces overfitting and improves generalization by aggregating decisions of individual trees.

To achieve the best performance, we optimized several RF hyperparameters: the number of trees (from 20 to 40), the maximum depth of each tree (from 5 to 10), and the criterion for measuring the quality of a split (Gini index or entropy). An RF classifier consisting of 30 trees with a maximum depth of 5 using the Gini index as the criterion for measuring the quality of the splits was employed during SFS.

#### 2.6.5. Artificial Neural Networks (ANNs)

Artificial neural networks are powerful tools for classification tasks, capable of learning complex relationships in data [25]. ANNs are constructed of processing units (neurons) that are interconnected. The connections between neurons are weighted, and these weights store the necessary information about the data. During the training process, weights are updated to minimize the difference between predicted and actual outputs using optimization techniques. We used a stochastic gradient descent technique with Adam optimization.

For optimizing training efficiency and achieving the best performance, we tested different values of mini-batch size and learning rate. The mini-batch size determines the number of training examples processed in each iteration, impacting both computational efficiency and gradient estimation quality. The mini-batch size was chosen from the set of values [4,  8,  16]. Meanwhile, the learning rate controls the step size of parameter updates during optimization, striking a balance between rapid convergence and stability. Learning rate was optimized from the set $\left[10^{-4},\;{\;10}^{-3},\;{\;10}^{-2},{\;10}^{-1}\right]$. During the SFS procedure, the mini-batch size was set to 8, and the learning rate was set to $10^{-3}$.

## 3. Results

**Figure 1:**
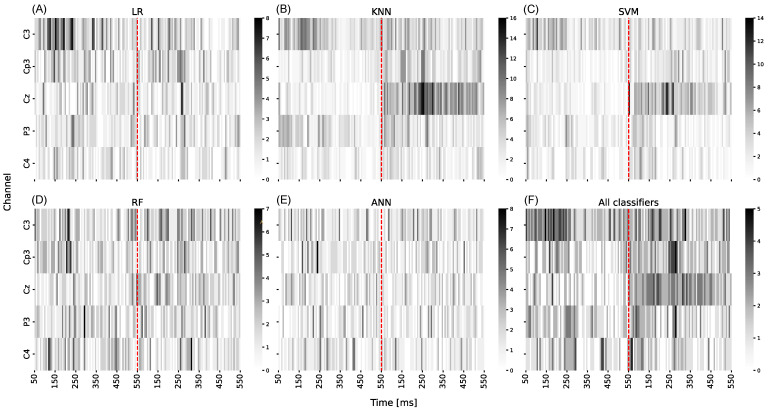
Feature maps following the feature selection process. Figures (**A**–**E**) represent the number of times each feature was selected across all subjects and tested values of $N_{avg}$, with darker colors indicating higher selection frequencies. Figure (**F**) shows the number of classifiers that selected each feature, where white indicates no selection and black indicates selection by all classifiers.

Figure 1 illustrates the feature maps resulting from the feature selection process using various classifiers. Figure 1A–E display the frequency with which each feature was selected across all subjects and all tested $N_{avg}$ values. In that way, the maximum number of times a feature can be selected was 90 (10 subjects × 9 values for $N_{avg}$).

Based on Figure 1A–E, it can be noticed that the most informative channels are C3 and Cz. Specifically, for channel C3, the interval from 100 to 250 ms exhibits the highest frequency of selected features, while for channel Cz, that interval is from 150 to 300 ms. Simpler classifiers, such as LR, KNN, and SVM, show more distinct intervals where most features are selected. In contrast, complex algorithms, such as RF and ANN, select features across the entire space. Figure 1F presents a map of features with information on how many classifiers have chosen said feature. It can be noticed that almost all classifiers show a higher concentration of selected features in the range from 100 to 300 ms for channels C3, Cp3, and Cz.

**Figure 2:**
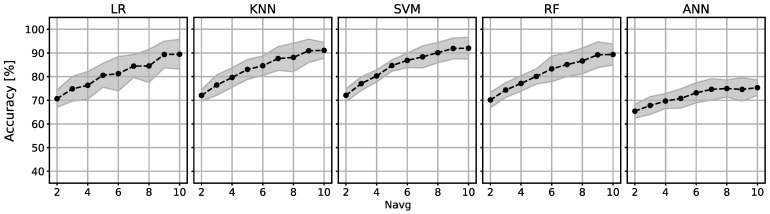
Accuracy of each classifier across all subjects in relation to the value $N_{avg}$. The dashed line represents the mean value of accuracy for all subjects, and the shaded area indicates the standard deviation interval.

The mean accuracy of each classifier across all subjects in relation to the value $N_{avg}$ can be seen in Figure 2. The ANN demonstrates underperformance compared to other classifiers, with the highest accuracy of 83.01% for $N_{avg}=10$, while the other classifiers manage to achieve maximal accuracies above 95% for $N_{avg}=10$. All classifier accuracies rise with higher values of $N_{avg}$. Accuracies for each classifier across subjects and $N_{avg}$ values are presented in Table 1.

**Figure 3:**
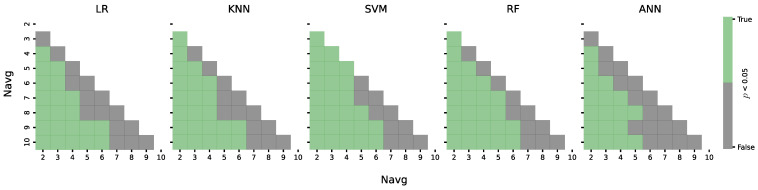
Intervals of statistical difference of each classifier across all subjects in relation to the value $N_{avg}$. Green squares represent intervals where there is a statistically significant difference, and black squares are in correlation with intervals with no significant difference.

Figure 3 shows intervals of statistically significant difference (p<0.05) between accuracies among all subjects for each classifier. It can be noticed that for all classifiers, accuracy can be significantly decreased when $N_{avg}\leq 6$ compared to $N_{avg}=10$. On the other hand, accuracy for $N_{avg}=2$ compared to all other values of $N_{avg}$ is significantly lower.

**Figure 4:**
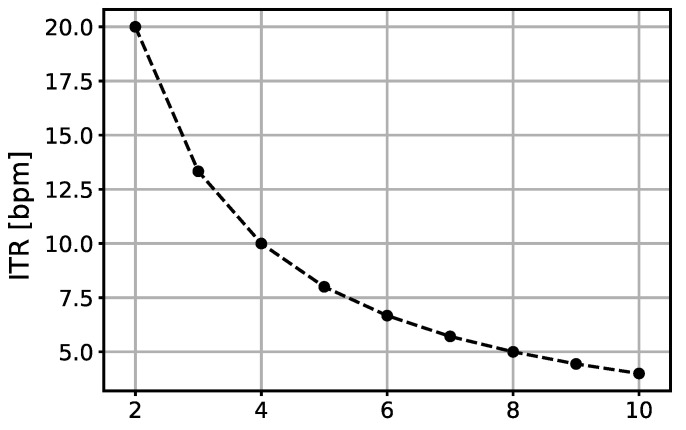
Information transfer rate in relation to $N_{avg}$.

Information transfer rate (ITR) is a metric useful for assessing the performance of a BCI system and is calculated as in [26] with the following parameters: (number of targets: 2, number of commands: 1, time in seconds per decision, which depends on $N_{avg}$ value). ITR quantifies the rate of information transfer per minute. Figure 4 presents how ITR is changing in relation to $N_{avg}$. It can be noticed that ITR gradually decreases with higher $N_{avg}$, with a maximum value of 20 bpm for $N_{avg}=2$ and a minimum value of 4 bpm for $N_{avg}=10$.

## 4. Discussion

This study investigated the feasibility of employing a multi-channel approach to improve the performance of the proposed BCI system. The BCI system uses sERP as a control signal and is based on a tactile attention task. This research focused on three primary aspects:

1. Finding the optimal subset of features: We implemented a sequential feature selection algorithm to extract the most important information from the signals. We extracted subsets of features that gave the highest accuracy during the selection process. The maximum size of the subset was set to 40 features. During the selection process, accuracy was obtained using 5-fold cross-validation.
2. Comparison of classification approaches: We compared the performance of five different machine learning algorithms. To achieve the best performance, we optimized the most important hyperparameters of each algorithm. Classification accuracy was utilized as a performance metric and was obtained through 5-fold cross-validation.
3. Testing the influence of α on BCI performance: We tested how the number of trials to be averaged influences the accuracy of classification and ITR. Parameter $N_{avg}$ was changed in the range from 2 to 10, and for each value, the previous two steps were conducted.

### 4.1. Optimal Subset of Features

To study the brain mechanisms related to evaluating stimuli, it is important to identify two distinct types of brain responses to sensory input: exogenous components and endogenous components. Exogenous components include early responses (N20, P40/50, N70) that are less affected by attention tasks during direct electrical stimulation of sensory pathways [16,27,28]. Endogenous components (P100, N140, P300) of longer latency, typically more than 100 ms, are influenced by cognitive tasks [29,30,31]. In this study, we narrowed our feature selection window to the interval 50–550 ms poststimulus since we aimed to include only the ERP components relevant to the task, as identified in previous studies.

During the feature selection process, several intervals were shown to be the most informative, meaning features from those regions were frequently selected. The LR classifier (Figure 1A) selected most features from the C3 channel in the 100–250 ms interval. Similar results were observed for the KNN classifier (Figure 1B), with intervals from 150 to 270 ms for the C3 channel and 170 to 300 ms for the CP3 channel. Figure 1C shows a similar pattern for the SVM classifier. These intervals comply with findings from the literature, confirming that the relevant features are related to endogenous sERP components that respond to attention tasks.

Based on the topography of sERP responses and the main sources of EEG activity expected in the contralateral somatosensory cortex, we selected a subset of EEG channels for our system [16,32]. Research [33] used eight EEG electrodes from the frontal and central regions: Fz, FC1, FC2, C3, Cz, C4, CP1, and CP2. In paper [34], 29 EEG channels were used, but the best ones for right index finger stimulation were FC3 and CP3.

In Figure 1F, the joint information of feature selection across all classifiers shows that EEG channels C4 and P3 have the fewest intervals where all classifiers selected features. These results indicate that the channels with the most-selected features are C3, CP3, and Cz. This further confirms that multi-channel features are relevant since our previous studies based on single-channel control favored the contralateral EEG channels. However, different intervals of feature selection at individual channels show that they carry distinct information about the cognitive task. Current results show that the combination of features from contralateral and central channels yielded superior performance.

Moreover, in our previous research [17], we examined differences between endogenous components for different attention tasks. The results showed a statistically significant difference in the 110–400 ms interval (N140, P3a, and P3b components) regarding amplitude and latency of the peaks, which is further supported by the findings in this paper, showing that the same intervals/channels that previously showed statistical differences between attended and unattended conditions now contain the most relevant features for automatic attention classification.

### 4.2. Influence of Classifier Selection on the BCI Performance

Table 1 represents accuracies for each tested classifier, for each sERP-averaging approach, and for each subject. The worst performance, over all subjects and all averaging approaches, was obtained by the ANN with an accuracy of 71.81 ± 5.21%. This can be attributed to the small dataset for this approach. This is also evident in Figure 1E, where all features had a small probability of being chosen, indicating the ANN’s inability to learn patterns from the given signals. The best performance was achieved by the SVM classifier (84.78 ± 7.50%), followed by the KNN classifier (83.68 ± 7.59%). The highest achieved accuracy across all subjects was 92.01 ± 4.88% for the SVM classifier with $N_{avg}=10$. For a single subject, the SVM classifier accuracy improved up to 98.95% for subject ID7 with nine averaged sERPs.

In study [35], the authors used a SVM classifier for SSSEP BCI control and selective tactile attention tasks, while the recognition was based on spatial-spectral features.

Review of other tactile BCI studies showed that linear discriminant analysis (LDA) is the most common method for classification in such systems [33,36,37]. This can be justified as LDA performs feature reduction before classification, extracting features that best separate the classes. However, because we used the SFS algorithm, LDA could not reach its full potential and was not tested. The advantage of SFS over LDA is that the maximum number of features after reduction is not limited, unlike LDA, where it must be less than the number of classes (in our case, two). In paper [33], an accuracy of 80% was achieved for binary classification with the LDA classifier. The same classifier was used for two-class problems in paper [36], where the achieved accuracy was above 90%. Compared to these papers, we managed to achieve better performance with the SFS methodology and different machine learning approaches.

### 4.3. Influence of $N_{avg}$ on the BCI Performance

BCI performance can be evaluated based on the accuracy of the decisions made and the time needed to make those decisions. A parameter that affects both evaluation metrics is the number of responses to individual stimuli for average ($N_{avg}$) to obtain the sERP. Higher values of $N_{avg}$ improve the signal-to-noise ratio (and consequently the accuracy) but prolong decision time. Therefore, it is important to find the optimal value of $N_{avg}$ that balances accuracy and decision time. We varied $N_{avg}$ from 2 to 10 to find the optimal performance. In BCI systems, the parameter that best describes the system’s speed is the ITR. For our BCI system, the number of targets is two, the number of commands is one, and the time in seconds per decision is $2\cdot N_{avg}\cdot \Delta t$.

Figure 2 shows that accuracy increases with higher values of $N_{avg}$ for each classifier. Figure 3 summarizes how much we can lower $N_{avg}$ without a significant difference in accuracy. For the LR classifier, a significant difference (*p* < 0.05) appears when $N_{avg}$ is lowered from 10 to 6. In that range, accuracy decreases from 89.26 ± 6.37% to 81.23 ± 7.72%, and ITR improves from 4 bpm to 6.67 bpm. This means that an 8% decrease in accuracy increases ITR by 60%. The KNN classifier shows similar results: a decrease of less than 10% in accuracy and a 60% increase in ITR is achieved if $N_{avg}$ is changed from 10 to 6. The ANN shows that $N_{avg}$ can be decreased even more without a significant drop in accuracy. However, because the overall performance of the ANN is poor, that drop will result in accuracies just above chance level, which are not useful for a BCI system with mean accuracies over 70%, even for $N_{avg}=2$. This allows for a significant increase in ITR in applications where speed of operation is crucial.

Table 2 presents the best performance for each classifier in the overall performance of the BCI system. It combines results from the previous discussion about finding the optimal value of $N_{avg}$ to balance accuracy and ITR. $N_{avg}$ is chosen as the first value that produced a statistically significant difference compared with $N_{avg}=10$.

These results are comparable to the performance reported in a very limited number of tactile ERP-based BCIs. Paper [12] reported an accuracy of 80% with an ITR of 2.9 bpm in their P300 BCI based on sERPs. Research [37] achieved a better ITR of 4.61–6.95 bpm, but with a drop in accuracy (64.5–75.5%). In paper [11], an accuracy of over 90% with an ITR of 6.88 bpm was reported for a BCI system based on electrical stimulation. However, this paper used a larger number of EEG channels (14 EEG channels), which enabled higher accuracy and higher complexity of the BCI system.

### 4.4. Multi-Channel Approach

In our previous research [18], we used single-channel BCI features, and the best performance (80.85% accuracy and an ITR of 4.29 bpm) was achieved with the SVM classifier and $N_{avg}=10$. The current study was oriented towards a multi-channel approach, where features were selected from one pool that includes information from five EEG channels. The multi-channel approach improved classification accuracy. For the SVM classifier and $N_{avg}=10$, the achieved accuracy was 92%. This allows more flexibility in choice of $N_{avg}$ parameter depending on the specific use-case scenario (i.e., accuracy vs. ITR trade-off). The multi-channel approach and advanced feature selection and classification have improved the overall performance of the system with mean accuracies over 70%, even for the lower $N_{avg}$ values in classification approaches.

## 5. Conclusions

In this study, we present an electrotactile BCI system based on sERPs elicited by electrical stimulation of the mixed nerves of the forearm with equal probability of stimulus occurrence. By employing a multi-channel approach in combination with sequential forward selection of features, we achieved significant improvements in classification accuracy and ITR. The optimal results are achieved by utilizing a SVM classifier, where accuracy was in the range from 88.08% to 97.78% across all subjects, for $N_{avg}=10$ (ITR = 4 bpm). The high accuracies achieved allow for a smaller number of averaged epochs for sERP generation and thus an increase in ITR. A statistically significant drop in accuracy occurred for $N_{avg}=6$ (ITR = 6.67 bpm) for the SVM classifier. For this value of $N_{avg}$, accuracy is in the range from 83.86% to 91.27% across all subjects. The proposed paradigm shows great potential for advancing tactile BCI applications, specifically in assistive devices for communication and control in severely disabled users, where the ITR and accuracy trade-off is a crucial issue depending on the specific use-case scenarios.

## Figures and Tables

**Table 1:** Classification performance for all subjects after selecting the optimal set of features for different classifiers and different numbers of averaged trials. Bolded values are associated with the highest accuracy for each classifier.

	$N_{avg}$	Accuracy [%]											
		ID1	ID2	ID3	ID4	ID5	ID6	ID7	ID8	ID9	ID10	MV ± STD	MV ± STD
**LR**	2	69.87	65.71	71.20	70.86	71.22	70.99	76.12	77.20	64.96	68.39	70.65 ± 3.89	81.24 ± 8.38
	3	71.66	67.38	79.41	71.13	76.95	76.99	80.35	83.33	66.69	74.65	74.85 ± 5.56	
	4	72.73	70.52	83.03	79.05	74.11	79.63	86.15	78.00	64.95	74.81	76.30 ± 6.20	
	5	78.25	77.80	83.85	77.65	81.96	86.48	83.38	89.21	71.97	75.06	80.56 ± 5.33	
	6	75.82	70.48	87.75	77.91	81.27	87.38	90.51	89.23	69.10	82.86	81.23 ± 7.72	
	7	80.74	80.47	93.15	81.01	87.17	84.31	84.29	92.73	78.18	82.29	84.43 ± 5.13	
	8	81.99	79.16	89.00	79.00	83.00	86.79	95.00	96.84	73.37	80.88	84.50 ± 7.40	
	9	88.97	82.35	90.92	80.92	90.98	89.80	98.18	97.57	82.48	91.32	89.35 ± 5.98	
	10	88.86	79.08	93.58	83.17	93.75	88.25	98.25	95.90	81.58	90.19	89.26 ± 6.37	
**KNN**	2	71.09	74.52	71.68	70.82	74.65	71.46	75.39	71.85	66.35	72.81	72.06 ± 2.58	83.68 ± 7.59
	3	72.03	78.24	77.65	76.38	76.21	76.27	80.37	84.81	67.46	75.05	76.45 ± 4.62	
	4	76.32	76.52	79.80	81.43	75.03	81.08	86.15	84.00	72.61	83.33	79.63 ± 4.37	
	5	85.32	82.75	84.40	80.75	81.96	80.36	92.19	86.11	75.02	81.51	83.04 ± 4.48	
	6	86.06	82.76	87.70	80.90	84.95	79.97	92.94	89.23	80.16	81.29	84.60 ± 4.37	
	7	79.78	82.17	91.52	87.03	88.88	90.40	94.29	94.55	80.71	87.01	87.63 ± 5.34	
	8	90.47	78.16	96.00	89.00	91.00	80.74	93.33	95.79	81.53	85.09	88.11 ± 6.40	
	9	90.29	84.63	93.27	91.05	92.22	87.19	96.35	96.32	82.55	91.54	90.54 ± 4.58	
	10	91.62	85.58	93.58	91.08	88.67	90.83	97.78	94.57	87.00	90.29	91.10 ± 3.61	
**SVM**	2	70.11	72.14	76.51	70.13	73.97	72.64	76.09	70.86	66.82	71.81	72.11 ± 2.92	84.78 ± 7.50
	3	76.11	73.89	79.04	75.36	78.36	80.94	80.37	75.56	71.28	79.49	77.04 ± 3.11	
	4	78.27	75.52	82.58	80.48	82.03	77.74	84.62	82.50	79.84	79.22	80.28 ± 2.72	
	5	82.08	88.88	88.00	81.35	85.58	82.78	86.33	82.92	83.56	85.28	84.68 ± 2.55	
	6	88.25	83.28	91.27	86.80	84.18	86.64	90.51	90.77	83.89	82.86	86.84 ± 3.25	
	7	84.20	81.19	91.38	87.90	89.75	84.23	97.14	93.64	84.31	89.74	88.35 ± 4.95	
	8	90.47	84.26	92.00	88.00	92.00	85.84	98.32	92.57	85.74	90.41	90.02 ± 4.31	
	9	89.19	89.26	96.54	88.50	93.33	90.65	98.95	97.57	86.08	87.87	91.68 ± 4.31	
	10	91.71	80.58	91.08	94.83	93.75	94.83	97.78	95.90	88.08	91.52	92.01 ± 4.88	
**RF**	2	70.11	70.24	73.74	65.99	72.37	70.29	74.28	71.12	62.64	70.59	70.14 ± 3.49	81.69 ± 7.81
	3	72.04	73.51	75.57	71.14	75.53	76.27	80.37	77.41	69.14	72.40	74.34 ± 3.32	
	4	75.73	77.93	77.87	70.00	76.41	78.14	83.85	78.50	74.56	78.21	77.12 ± 3.50	
	5	77.68	78.45	83.81	76.56	79.50	80.97	85.33	82.82	74.36	81.51	80.10 ± 3.43	
	6	81.20	76.52	86.30	77.12	80.61	83.62	95.15	89.23	79.39	83.60	83.27 ± 5.73	
	7	83.51	79.57	89.67	80.25	87.97	80.59	94.29	90.91	78.97	85.19	85.09 ± 5.39	
	8	82.98	80.21	90.00	86.00	90.00	84.74	96.67	92.63	77.74	85.15	86.61 ± 5.77	
	9	88.09	84.71	92.16	83.99	92.16	84.97	98.00	98.75	82.61	86.76	89.22 ± 5.79	
	10	87.81	84.08	92.33	83.25	85.83	89.42	95.78	94.67	85.75	94.48	89.34 ± 4.68	
**ANN**	2	63.97	66.90	66.37	63.41	65.90	65.11	71.28	67.73	59.34	63.94	65.40 ± 3.13	71.81 ± 5.21
	3	66.82	65.60	71.74	66.20	69.24	65.84	73.03	72.96	60.29	66.03	67.78 ± 3.99	
	4	68.63	67.10	72.69	66.67	68.87	72.86	73.85	73.50	63.94	68.69	69.68 ± 3.38	
	5	70.50	67.37	73.46	65.70	74.12	72.86	72.57	75.91	62.22	73.08	70.78 ± 4.32	
	6	73.48	69.03	79.71	71.35	71.16	72.36	76.18	79.23	64.81	74.09	73.14 ± 4.53	
	7	73.25	72.25	80.18	70.65	76.81	71.15	78.57	82.73	67.39	73.25	74.62 ± 4.79	
	8	71.29	72.74	77.00	75.00	77.00	73.63	78.33	81.87	68.21	74.50	74.96 ± 3.85	
	9	72.06	70.37	76.34	68.04	78.63	74.18	81.27	83.01	67.58	74.49	74.60 ± 5.29	
	10	75.14	73.50	77.17	71.42	79.58	75.08	76.44	81.24	69.83	73.81	75.32 ± 3.48	

MV = mean value, STD = standard deviation.

**Table 2:** Performance for each classifier for optimal $N_{avg}$.

Classifier	$\mathrm{Optimal}\;N_{avg}$	Optimal Accuracy [%] MV ± STD	Optimal ITR [bmp]
LR	6	81.23 ± 7.72	6.67
KNN	6	84.60 ± 4.37	6.67
SVM	6	86.84 ± 3.25	6.67
RF	6	83.27 ± 5.73	6.67
ANN	5	70.78 ± 4.32	8

MV = mean value, STD = standard deviation.

## Data Availability

The data supporting the findings of this study will be made available by the corresponding author upon reasonable request.
